# RETRACTION: UBE2C Induces Cisplatin Resistance via ZEB1/2‐Dependent Upregulation of ABCG2 and ERCC1 in NSCLC Cells

**DOI:** 10.1155/jo/9863927

**Published:** 2025-12-12

**Authors:** Journal of Oncology

RETRACTION: Y. Wu, D. Jin, X. Wang, et al., “UBE2C Induces Cisplatin Resistance via ZEB1/2‐Dependent Upregulation of ABCG2 and ERCC1 in NSCLC Cells,” *Journal of Oncology*, 2019, 8607859, https://doi.org/10.1155/2019/8607859.

The above article, published online on 01 January 2019 in Wiley Online Library (https://wileyonlinelibrary.com), and its correction (https://onlinelibrary.wiley.com/doi/10.1155/2020/5025641), has been retracted by John Wiley & Sons Ltd. The retraction has been agreed due to multiple concerns with the figures, some of which have been raised on PubPeer [1]. Investigation of the concerns identified the following issues:

• In Figure 1(a):
  ◦ The UBE2C bands appear similar to the GAPDH bands
  ◦ The ZEB1 bands appear similar to the GAPDH bands in Figure 3(i)
  ◦ The ZEB1 bands appear similar to the GAPDH bands in Figure 4(a)
  ◦ The ZEB2 bands appear similar to the GAPDH bands
  ◦ The ZEB2 bands appear similar to the GAPDH bands in Figure 4(a)
  ◦ The ZEB1 WB bands appear similar to the Tubulin WB in Figure 4(a) when flipped horizontally
• In Figure 1(c), the A549/UBE2C bands appear identical to the A59/ZEB2 bands
• In Figure 1(d), the H1299/ZEB2 WB bands appear identical to the A59/ERCC1 WB bands shown in Figure 1(k)
• In Figure 1(d), the H1299/Tubulin WB bands appear identical to the Tubulin WB bands shown in Figure 3(e) and the A549/Tubulin WB bands in Figure 1(k)
• In Figure 1(g), the first two A549/Tubulin WB bands appear similar to the first two Tubulin WB bands shown in Figure 3(d)
• In Figure 1(k), the A549/Tubulin WB appear similar to the Tubulin WB bands shown in Figure 3(e)
• In Figure 2(b), the A549/UBE2C bands appear identical to the H1299/ZEB1 bands
• In Figure 5(a), the UBE2C image appears similar to the Control image in Figure 5(h) of [2], when rotated 180°
• In Figure 5(d):
  ◦ The Invasion/PBS image appears similar to the Migration/Control image shown in Figure 5(i) of [2].
  ◦ There are repeated elements between the Invasion/PBS panel and the Invasion/control panel
  ◦ There are repeated elements between the Migration/Si UBE2C panel and the Migration/UBE2C + siZEB1 panel
• In Figure 5(e):
  ◦ The left‐hand UBE2C bands appear similar to the right‐hand UBE2C bands
  ◦ The left‐hand ZEB1 bands appear similar to the right‐hand Vimentin bands
  ◦ The left‐hand E‐Cadherin bands appear similar to the right‐hand E‐Cadherin bands

The results are therefore deemed unreliable, and the article is retracted.

Jiwei Guo agrees to the retraction, but not the wording of the notice. The remaining authors were unresponsive.
